# Conservative Management of an Anterior Maxillary Calcifying Epithelial Odontogenic Tumor With Customized Titanium Mesh-Guided Bone Regeneration and Implant Rehabilitation

**DOI:** 10.7759/cureus.111467

**Published:** 2026-06-25

**Authors:** Axelle Dumoulin, Mathilde Natu, Sophie Lejeune, Julien Hamon

**Affiliations:** 1 Oral Surgery, University Hospital Center, Rennes, FRA

**Keywords:** calcifying epithelial odontogenic tumor, dental implant, guided bone regeneration, implant rehabilitation, pindborg tumor, titanium mesh

## Abstract

Calcifying epithelial odontogenic tumor (CEOT), or Pindborg tumor, is a rare benign odontogenic neoplasm with a recognized risk of local recurrence. Management of anterior maxillary lesions is particularly challenging due to aesthetic and functional implications. We report the case of a 26-year-old woman who presented with a missing maxillary right canine. Cone beam computed tomography (CBCT) revealed a 2.2 × 1.5 × 1.2 cm, well-defined mixed lesion associated with an impacted tooth 13 with internal calcifications. Incisional biopsy suggested CEOT. Following a multidisciplinary discussion, a conservative surgical approach was selected. Enucleation with curettage and extraction of tooth #13 were performed. Histopathology confirmed CEOT. Complete bone healing was observed at eight months. Secondary reconstruction using guided bone regeneration with a customized titanium mesh was performed, followed by implant placement and prosthetic rehabilitation. At the 30-month follow-up, no evidence of recurrence or implant-related complications was observed. In effect, in selected anterior maxillary CEOTs, conservative surgery followed by staged reconstruction and implant rehabilitation may achieve satisfactory oncologic, functional, and aesthetic outcomes.

## Introduction

Calcifying epithelial odontogenic tumor (CEOT), also known as Pindborg tumor, is a rare, benign odontogenic neoplasm accounting for approximately 1% of odontogenic tumors [1]. It typically presents as a slow-growing, painless swelling, most commonly in the posterior mandible of middle-aged adults, and is frequently associated with an impacted tooth [2]. Radiographically, CEOT appears as a well-defined unilocular or multilocular radiolucency with characteristic internal calcifications, described as "driven snow" opacities [2].

Although most cases arise in the posterior mandible, maxillary locations are less frequent and present specific therapeutic challenges due to aesthetic and reconstructive constraints [2].

CEOT is considered benign but may exhibit locally aggressive behavior, with reported recurrence rates ranging from 10% to 15%, particularly after conservative treatment and in maxillary locations [3,4]. Wide excision has traditionally been recommended; however, treatment is often individualized based on lesion size, location, radiographic margins, cortical involvement, histologic features, and recurrence risk. Such radical approaches may be highly mutilating in the anterior maxilla, especially in young patients.

In carefully selected cases, characterized by small lesion size, well-defined radiographic borders, absence of cortical perforation or soft-tissue extension, aesthetic-zone location, and favorable patient compliance, conservative surgery may preserve tissue architecture and facilitate subsequent implant rehabilitation [5,6].

This report describes the successful conservative management of an anterior maxillary CEOT in a young patient, followed by staged guided bone regeneration and implant rehabilitation, with emphasis on selection criteria, surgical technique, reconstructive approach, and long-term outcomes.

This report follows the SCARE 2023 guidelines for surgical case reports [7].

## Case presentation

Patient information

A 26-year-old woman with no relevant medical history was referred for a missing maxillary right canine. The patient was asymptomatic.

Clinical findings

Clinical examination revealed the absence of tooth #13 (FDI (Fédération Dentaire Internationale) notation: maxillary right canine), associated with diastemas and a vestibular fistula. There was no pain, facial swelling, paraesthesia, or palpable lymphadenopathy. No nasal symptoms or functional disturbance were reported. Adjacent teeth (#12 and #14) responded positively to thermal and electric pulp testing, confirming vitality. Periodontal probing depths around adjacent teeth were within normal limits.

Diagnostic assessment

Cone beam computed tomography (CBCT) revealed a 2.2 × 1.5 × 1.2 cm, well-defined mixed lesion associated with impacted tooth #13 (FDI), with internal calcifications and no evidence of root resorption or cortical perforation (Figure 1).

**Figure 1:**
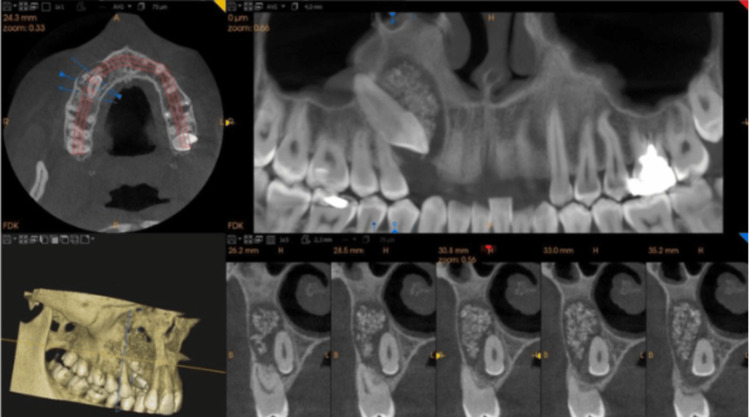
Preoperative CBCT imaging showing a 2.2 × 1.5 × 1.2 cm, well-defined mixed lesion associated with impacted tooth #13, with internal calcifications, in the anterior maxilla CBCT: cone beam computed tomography

Incisional biopsy demonstrated sheets and islands of polyhedral epithelial cells with abundant eosinophilic cytoplasm and distinct cell borders. Extracellular deposits of amyloid-like eosinophilic material were observed, containing characteristic concentric calcifications (Liesegang rings). The surrounding stroma was fibrous. No features of malignancy were identified. These findings were consistent with calcifying epithelial odontogenic tumor (Figure 2) [8]. Differential diagnoses included odontoma and calcifying odontogenic cyst.

**Figure 2:**
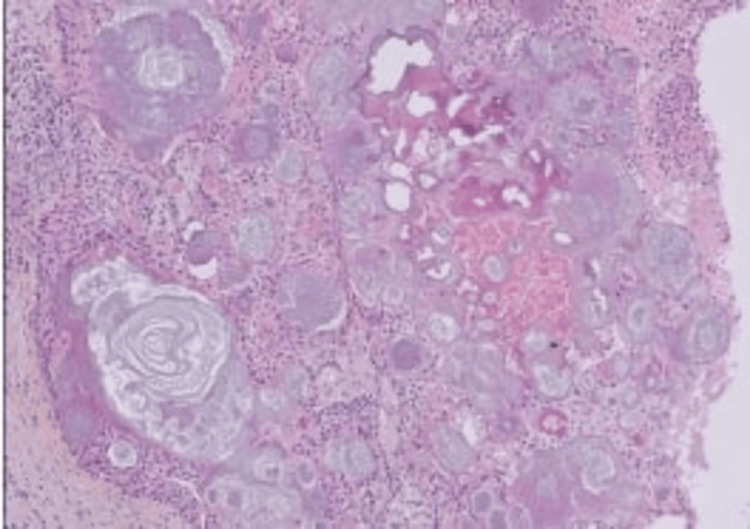
Histopathological sections with hematoxylin and eosin staining, original magnification ×100

Therapeutic intervention

Following a multidisciplinary discussion, a conservative surgical approach was selected because the lesion demonstrated favorable characteristics for tissue-preserving management: the patient was compliant and suitable for long-term follow-up, and the lesion size was limited, with well-defined margins. Moreover, there was no evidence of cortical perforation, and no root resorption was observed. Radical surgery would have resulted in significant aesthetic and functional morbidity. The patient was informed about the potential risk of recurrence associated with conservative treatment and the necessity of long-term clinical and radiographic surveillance. Written informed consent was obtained.

Surgery was performed under local anesthesia. A mucoperiosteal flap was elevated to expose the lesion. Adjacent teeth (#12 and #14) were confirmed vital preoperatively and were preserved. Tooth #13 (FDI) was extracted along with the associated lesion (Figure 3). Complete enucleation of the tumor was performed with careful dissection from the surrounding bone. Rotary curettage of the cavity was then performed using a low-speed round bur under copious irrigation to eliminate any residual tumor tissue (Figure 4). Completeness of excision was assessed intraoperatively by direct visual inspection and palpation of the bony walls for any residual soft tissue. The surgical specimen was submitted in its entirety for histopathological examination, which confirmed the diagnosis of calcifying epithelial odontogenic tumor.

**Figure 3:**
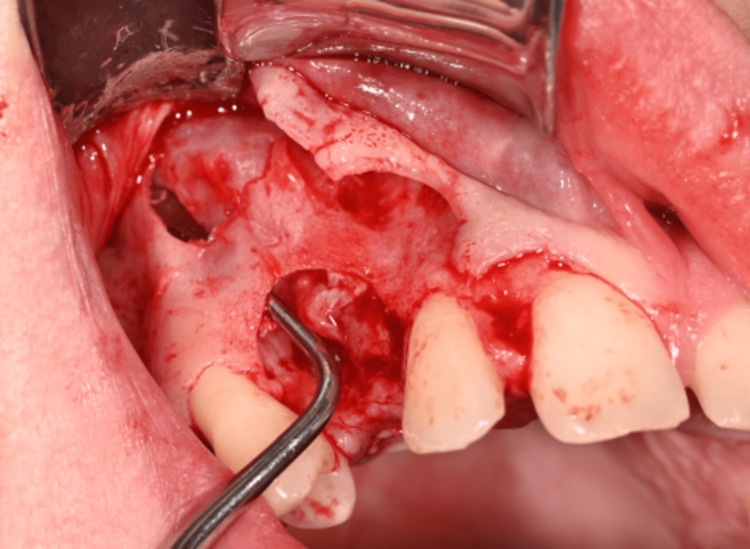
Intraoperative view during enucleation of the lesion after extraction of tooth #13

**Figure 4:**
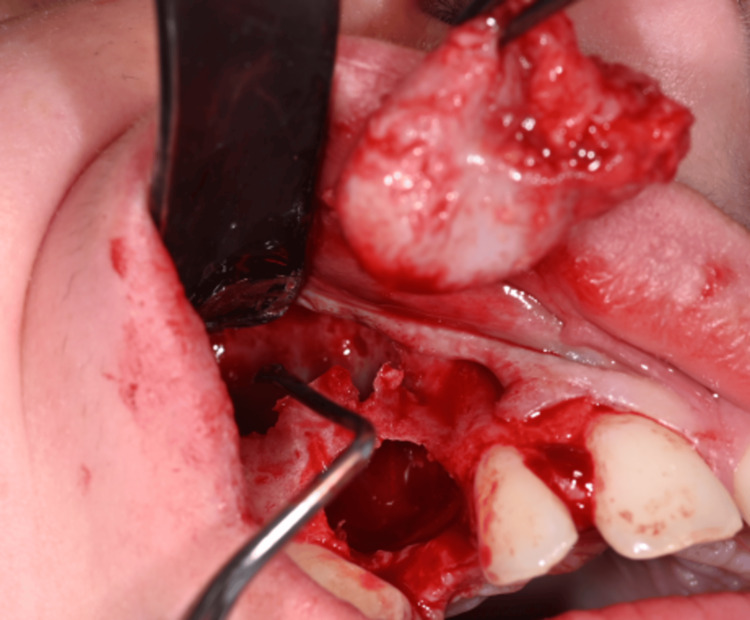
Intraoperative view following complete enucleation of the surgical cavity

Follow-up and outcomes

CBCT at eight months showed complete bone healing with no residual lesion. The residual ridge width was 2.9 mm.

After local disease stabilization, reconstruction was performed using guided bone regeneration with a customized titanium mesh (Yxoss® - ReOss®, Filderstadt, Germany) combined with xenograft and allograft. After eight months of healing, CBCT demonstrated satisfactory bone regeneration with a ridge width of 7.6 mm, corresponding to a horizontal bone gain of 4.7 mm (Figure 5).

**Figure 5:**
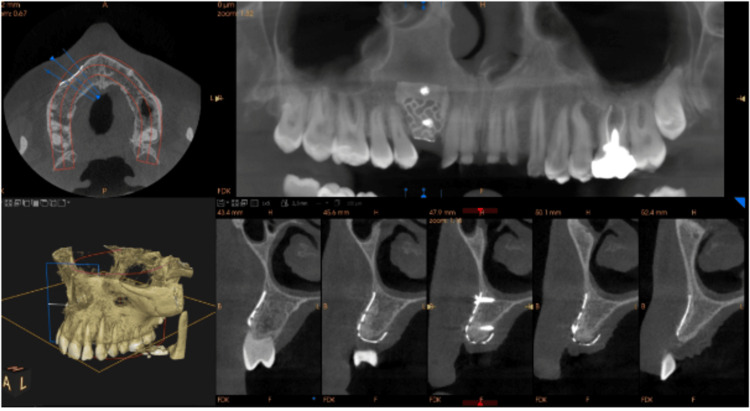
Customized titanium mesh (Yxoss® – ReOss®) in place eight months after the guided bone regeneration procedure

Implant placement was performed using a prosthetically driven guided approach. A 3.5 × 11.5 mm implant was placed with an insertion torque of 25 N/cm. Provisional restoration was maintained for six months to allow soft tissue conditioning and emergence profile optimization.

Clinical and radiological follow-up was conducted. At 30 months following tumor removal, a periapical radiograph showed no peri-implant radiolucency and minimal marginal bone loss limited to the first thread (Figure 6). Peri-implant probing depths were within normal limits (≤3 mm) with no bleeding on probing. No evidence of recurrence or implant-related complications was observed.

**Figure 6:**
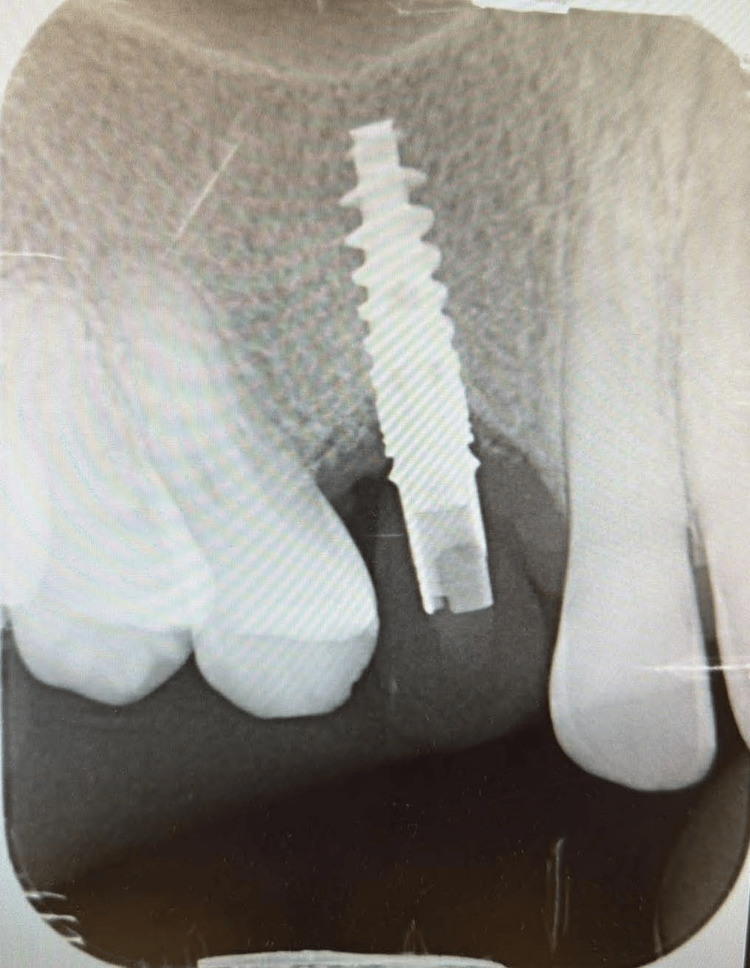
Periapical radiograph at the 30-month follow-up showing the implant in position #13 (FDI) with provisional restoration

Timeline

An initial consultation was performed, followed by CBCT imaging for radiological assessment of the lesion. An incisional biopsy was then carried out, which confirmed the diagnosis of calcifying epithelial odontogenic tumor. Based on the clinical, radiological, and histopathological findings, conservative surgery, including enucleation, curettage, and extraction of tooth #13 (FDI), was performed. An eight-month radiological follow-up confirmed complete bone healing. Secondary reconstruction was subsequently achieved using guided bone regeneration with a customized titanium mesh. An additional eight-month radiological control demonstrated satisfactory bone regeneration, allowing implant placement using a prosthetically driven guided approach. The six-month clinical and radiological follow-up confirmed successful provisional prosthetic rehabilitation, followed by definitive prosthetic rehabilitation after further clinical and radiological validation. Annual clinical and radiological follow-up was maintained thereafter. Table 1 summarizes the timeline for this patient.

**Table 1:** Summary of the timeline CBCT: cone beam computed tomography; FDI (Fédération Dentaire Internationale) notation: maxillary right canine; CEOT: calcifying epithelial odontogenic tumor

Timepoint	Procedure / Event	Outcome
Initial consultation	Clinical examination, CBCT	2.2 × 1.5 × 1.2 cm mixed lesion with impacted tooth #13 (FDI)
	Incisional biopsy	Histopathology suggestive of CEOT
	Conservative surgery: enucleation, curettage, extraction of tooth 13 (FDI)	Complete macroscopic removal; CEOT confirmed on final histopathology
Month 8	CBCT follow-up	Complete bone healing, no residual lesion; ridge width 2.9 mm
	Guided bone regeneration (customized titanium mesh + xenograft/allograft)	Uneventful healing
Month 16	CBCT follow-up	Ridge width 7.6 mm (horizontal gain +4.7 mm; vertical gain +6 mm)
	Implant placement: 3.5 × 11.5 mm, 25 N/cm	Primary stability achieved
Month 22	Provisional prosthetic restoration	Soft tissue conditioning initiated
Month 28	Definitive prosthetic rehabilitation	Functional and aesthetic outcome achieved
Month 30	Clinical and radiographic follow-up	No recurrence; minimal marginal bone remodeling; probing depths ≤3 mm

## Discussion

This case highlights the therapeutic dilemma between oncologic safety and preservation of anterior maxillary structures in young patients with CEOT. Although CEOT is a benign tumor, it may demonstrate locally aggressive behavior with reported recurrence rates, particularly following conservative treatment and in maxillary locations [3,4]. Consequently, wide surgical excision has historically been recommended. However, radical resection in the anterior maxilla can result in significant aesthetic and functional impairment, complicating prosthetic rehabilitation.

The decision to proceed with conservative treatment was based on a careful risk-benefit analysis. Several factors supported this approach: (1) the lesion was small (2.2 × 1.5 × 1.2 cm), (2) radiographic borders were well-defined, (3) there was no cortical perforation or soft-tissue extension, (4) no root resorption of adjacent teeth was observed, and (5) the patient was young, compliant, and committed to long-term surveillance. Under these conditions, enucleation combined with curettage may provide adequate local control, as supported by previous reports [3,4,9-13]. Radical resection in the anterior maxilla would have resulted in significant aesthetic disfigurement, loss of alveolar bone and adjacent teeth, nasal floor involvement, and complex reconstructive requirements (e.g., microvascular free flap). Given the benign nature of CEOT and the favorable lesion characteristics, such morbidity was considered disproportionate.

Reported recurrence rates for CEOT range from 10% to 15% overall [3,4]. However, recurrence is more frequently associated with large lesions, ill-defined margins, cortical perforation, and incomplete excision rather than the surgical modality per se. Chrcanovic and Gomez analyzed 339 reported cases and found that recurrence was primarily related to incomplete removal rather than the type of surgical approach [14]. Ai-Ru et al. reported no recurrence after enucleation in nine cases with well-defined borders [5]. Morais et al. described successful conservative management with long-term follow-up [9]. Kim et al. and Singh et al. similarly reported favorable outcomes after enucleation and curettage in maxillary CEOT [10,11]. These findings support that conservative treatment may be oncologically safe in carefully selected cases, provided long-term surveillance is maintained. CEOT recurrence has been reported to occur between 1 and 15 years after initial treatment, with a mean time to recurrence of approximately 5 years [3,4]. Risk factors for recurrence include large lesion size, ill-defined radiographic margins, cortical perforation, incomplete excision, and maxillary location [4]. Given these considerations, long-term surveillance is mandatory.

In the present case, the following surveillance protocol was implemented: clinical examination every 3 months during the first year, then every 6 months for years 2-5, and annually thereafter. Radiographic surveillance at each visit and CBCT at 6 months, 12 months, 24 months, and then every 2 years or if clinical suspicion arises. This protocol is consistent with published recommendations for conservative management of benign odontogenic tumors [3,4,9]. At the 30-month follow-up, no evidence of recurrence was observed, supporting the validity of this approach in carefully selected cases.

The reconstructive phase represents a key aspect of this report. Few studies describe implant rehabilitation following CEOT management. The use of a patient-specific titanium mesh allowed precise three-dimensional bone regeneration and optimal implant positioning, which would have been difficult to achieve with conventional techniques alone [5,6]. This staged approach demonstrates that local disease control and prosthetic rehabilitation can be successfully integrated, potentially improving long-term functional and aesthetic outcomes compared with radical resection. This case supports that, under strict selection criteria, conservative management may represent a reliable alternative to radical resection in anterior maxillary CEOT. However, this approach remains highly case-dependent and should not be generalized without strict radiological and clinical selection criteria.

Limitations include the single-case design, limited iconographic documentation of the reconstructive procedures, and the need for long-term surveillance, as late recurrence remains possible. In addition, the findings cannot be readily generalized, as patient-specific anatomical and therapeutic factors may have influenced the outcome. Functional and aesthetic outcomes were not assessed using standardized, validated measures, and patient-reported outcomes were not systematically collected.

Future work should focus on prolonged follow-up, more standardized reconstructive procedures, and systematic evaluation of functional, aesthetic, and patient-reported outcomes. Because of the rarity of this presentation, multicenter collaboration and pooled analyses would be valuable to strengthen the evidence base and refine therapeutic and reconstructive decision-making.

## Conclusions

Conservative surgery may be considered in selected anterior maxillary CEOTs after careful clinical, radiographic, histopathologic, and patient-related assessment. This approach remains highly case-dependent and should not be generalized without strict selection criteria, including small lesion size, well-defined radiographic margins, absence of cortical perforation, and a compliant patient suitable for long-term surveillance. In the present case, strict selection criteria allowed tissue preservation while maintaining local disease control. Staged implant rehabilitation using guided bone regeneration with a customized titanium mesh achieved a horizontal bone gain of 4.7 mm and a vertical bone gain of approximately 6 mm, enabling successful implant placement. At 30 months following tumor removal, functional and aesthetic rehabilitation was achieved with no clinical or radiographic evidence of recurrence and healthy peri-implant soft tissues. 

Long-term surveillance remains mandatory, given the recognized risk of delayed recurrence, which has been reported up to 15 years after initial treatment. Further studies with larger sample sizes and longer follow-up periods are needed to validate conservative management protocols for anterior maxillary CEOT.
